# The Interactive Effect of Rainfall and Nitrogen Deposition on Soil Respiration and Its Components in a Temperate Forest Ecosystem

**DOI:** 10.3390/plants15091340

**Published:** 2026-04-28

**Authors:** Ghani Subhan, Ziyuan Wang, Fuqi Wen, Wenxing Luo, Meiping Chen, Xiaoyi Shen, Yanbin Hao

**Affiliations:** 1 College of Life Sciences, University of Chinese Academy of Sciences, Beijing 100049, China; ghani.ucas893@mails.ucas.ac.cn (G.S.); wangziyuan23@mails.ucas.ac.cn (Z.W.); wenfuqi19@mails.ucas.ac.cn (F.W.); luowenxing20@mails.ucas.ac.cn (W.L.); chenmeiping23@mails.ucas.ac.cn (M.C.); shenxiaoyi22@mails.ucas.ac.cn (X.S.); 2 Beijing Yanshan Earth Critical Zone National Research Station, University of Chinese Academy of Sciences, Beijing 101408, China; 3 University of Chinese Academy of Sciences, Beijing 100049, China

**Keywords:** nitrogen addition, drought, precipitation change, autotrophic respiration, heterotrophic respiration, temperate forest

## Abstract

Rising human-caused nitrogen (N) deposition and increased rainfall variability threaten the capacity of temperate forests to sequester carbon. However, the combined effects of N enrichment and moisture changes on total soil respiration (Rs), including its autotrophic (Ra) and heterotrophic (Rh) components, remain poorly understood, especially in northern China’s warm-temperate forests. To explore this, a factorial field experiment was conducted at the Beijing Yanshan Earth Critical Zone National Research Station in Huairou District, Beijing. The experiment involved N addition (50 kg N ha^−1^ yr^−1^ as urea [CO(NH_2_)_2_]) and precipitation manipulation (±50% of ambient throughfall) during the 2024 growing season. Six treatments were implemented: control (CK), nitrogen addition (NA), 50% increased precipitation (W+50%), 50% decreased precipitation (W−50%), nitrogen addition with increased precipitation (NW+50%), and nitrogen addition with decreased precipitation (NW−50%). Under natural rainfall conditions, N addition increased Rs (+11.8%; *p* < 0.05). However, the effects of N largely depended on water availability: with increased rainfall, N addition significantly boosted Rs, Rh, and Ra by promoting fine root biomass and accelerating litter decomposition; under reduced rainfall, N addition still increased Rs, Rh, and Ra compared to drought alone (NW−50% vs. W−50%), though the extent of stimulation was considerably lower than under elevated precipitation, indicating that water availability influences the strength of N effects on forest soil respiration. Structural equation modelling (SEM; χ^2^/df = 1.8, RMSEA = 0.040, CFI = 0.97) revealed that water availability was a key mediator of the interaction between N addition and precipitation. These findings enhance understanding of how nitrogen supply and water availability interact in temperate forest soils, though further validation across other forest types and over longer periods remains necessary.

## 1. Introduction

Temperate forest ecosystems cover about one-third of Earth’s land surface and are important terrestrial carbon (C) sinks. Their capacity to sequester carbon is increasingly threatened by rising atmospheric nitrogen (N) deposition and more variable rainfall [1,2]. A key component of the C budget in these forests is soil respiration (Rs), which includes CO_2_ release from both autotrophic root respiration (Ra) and heterotrophic microbial decomposition (Rh). This process is the second-largest terrestrial C flux globally, releasing roughly ten times more carbon annually than human activities [3]. Importantly, the responses of Rs, Ra, and Rh to N enrichment depend on water availability: water acts as a switch, determining whether nitrogen addition stimulates growth-mediated microbial activity and root function, or causes stress by increasing osmotic pressure and accumulating toxic inorganic N ions, mostly ammonium (NH_4_^+^) and nitrate (NO_3_^−^) at elevated concentrations [4,5]. While there is substantial evidence on how N deposition or rainfall alone affects Rs in temperate forests, the combined effects of nitrogen supply and water availability on total soil respiration and its components remain poorly understood, especially in the warm-temperate forests of northern China [4,6]. Filling this knowledge gap is crucial for accurately predicting the carbon sequestration potential of these ecosystems amid accelerating global change.

Globally, atmospheric nitrogen (N) deposition has increased due to fossil fuel burning and agricultural intensification, changing the nutrient levels in forest soils [2]. However, how Rs responds to this N increase varies greatly depending on the specific context. In ecosystems limited by N, adding nitrogen can boost Rs by reducing nutrient limitations on plant growth (Ra) and boosting microbial activity (Rh) [4]. On the other hand, recent long-term experiments and meta-analyses show that in forests nearing N saturation, chronic N input frequently suppresses Rs [7,8]. This suppression is often attributed to mechanisms such as soil acidification, which induces aluminum toxicity and inhibits microbial enzyme activity, or to the “microbial efficiency” hypothesis, in which N addition suppresses the decomposition of recalcitrant organic matter by shifting fungal communities [7,9]. For instance, Frey et al. [8] demonstrated that increased N availability suppressed annual Rs in mixed temperate forests regardless of acidification, primarily by reducing root-associated respiration. Forsmark et al. [9] similarly found that nitrogen deposition altered microbial communities, diverting them from taxa proficient in degrading complex organic matter, thereby promoting soil carbon accumulation. These contrasting findings highlight the complexity of “ecosystem specificity” and the necessity of disentangling the abiotic and biotic drivers, such as soil pH and microbial biomass, controlling the direction and magnitude of the N effect on soil respiration.

In conjunction with N deposition, climate change is reshaping global precipitation patterns, leading to changes in rainfall frequency and intensity [10]. Soil respiration is highly sensitive to soil moisture, typically following a parabolic relationship, with activity peaking at optimal moisture levels and decreasing under drought or waterlogging conditions [10,11]. Recent global syntheses indicate that changes in precipitation can surpass temperature effects in regulating soil C [10]. Importantly, the interaction between N deposition and precipitation is often non-additive, meaning the effect of N on Rs depends on water availability. Water availability often acts as a “switch,” determining whether N functions as a fertiliser or a stressor. For example, N addition may increase Rs during wet periods by promoting vigorous plant and microbial growth but suppress it during drought by increasing osmotic stress or causing the buildup of toxic inorganic N ions, principally (NH_4_^+^) and (NO_3_^−^), at elevated concentrations [4,5]. Water availability influences N-induced soil respiration by controlling substrate diffusion to microbial cells, soil enzyme activity, and root metabolic rates: under sufficient moisture, added N stimulates Rs by alleviating nutrient constraints on both Ra and Rh, whereas during drought, reduced soil water limits microbial substrate supply and root function, reducing or negating N-driven stimulation [4,5]. Field evidence further suggests that this moisture-dependent modulation differentially affects the component fluxes of Rs, with Rh showing greater sensitivity to the N × water interaction than Ra [6,12]. Whether these interaction patterns persist under factorial N + precipitation manipulation in warm-temperate oak-dominated forests of northern China has not yet been tested experimentally.

To address these gaps, we conducted a factorial field experiment in a temperate forest in North China, manipulating nitrogen (N) addition and precipitation to assess their effects on Rs and its autotrophic and heterotrophic components. This study aimed to (1) examine the individual and combined impacts of N addition and precipitation changes on Rs, Ra, and Rh; and (2) investigate how these treatments influence the relationships between respiration components and key environmental factors, including soil water content, temperature, pH, microbial biomass, and fine root biomass. Based on these objectives, we proposed the following hypotheses: (i) N addition would increase Rs under both ambient and elevated precipitation, but the stimulatory effect would be diminished during drought conditions due to moisture-dependent limitations on microbial and root activity; and (ii) heterotrophic respiration (Rh) would primarily drive variations in Rs because of its greater sensitivity to soil water and pH compared with autotrophic respiration (Ra).

By simultaneously measuring total soil respiration and its autotrophic and heterotrophic components under factorial nitrogen and precipitation treatments, this study provides evidence on how water availability affects nitrogen impacts on soil carbon fluxes in a warm-temperate forest of northern China.

## 2. Materials and Methods

### 2.1. Study Site Description

The study was conducted at the Beijing Yanshan Earth Critical Zone National Research Station in Huairou District, Beijing, China (40°25′0.84″ N, 116°39′14.76″ E; 140 m above sea level) (Figure S1). The vegetation includes mixed communities of trees, shrubs, herbs, and vines typical of North China, with dominant tree species such as *Quercus mongolica*, *Pinus tabuliformis*, *Quercus acutissima*, *Populus* spp., *Tilia cordata*, and *Acer* spp. The region has a warm-temperate, semi-humid monsoon climate with hot, humid summers and cold, dry winters. About 88% of the annual precipitation falls between June and September. From 1978 to 2020, the average yearly rainfall was 544.3 mm, and during 2020–2021, the average annual air temperature was 12.4 °C [13]. The soil is classified as Chromic Cambisol, with a pH of 7.4 ± 0.4 (0–10 cm).

### 2.2. Experimental Design, Soil Sampling, and Litter-Bag Experiment

Nine 20 m × 20 m plots were established, each divided into two 10 m × 20 m subplots, resulting in a total of 18 subplots. Six treatments were randomly assigned with three replicates each: control (CK), nitrogen addition (NA), 50% increased precipitation (W+50%), 50% reduced precipitation (W−50%), nitrogen addition plus 50% increased precipitation (NW+50%), and nitrogen addition plus 50% reduced precipitation (NW−50%). Within each 10 m × 20 m subplot, one designated trench zone (60 cm wide × 1 m deep) was established in December 2023, while the adjacent area of the same subplot remained completely undisturbed. PVC collars installed in the undisturbed zone of each subplot were used to measure total soil respiration (Rs), while collars within the trenched zone were used to measure heterotrophic respiration (Rh). Autotrophic respiration (Ra) was calculated as Ra = Rs − Rh. Nitrogen addition and precipitation treatments started in April 2023, with the growing season (April–September 2023) serving as a treatment-establishment period [14]. No soil respiration or litter decomposition data were collected during 2023, as this period was solely for establishing treatments and allowing trench-induced root decomposition effects to subside. All gas flux and litter data used in statistical analyses stemmed from the 2024 growing season (April–September 2024).

Nitrogen was applied as urea at 50 kg N ha^−1^ yr^−1^. Urea was dissolved in 20 L of water per subplot and sprayed monthly from April to September using an electric sprayer held approximately 50 cm above the soil surface. Each subplot was sprayed with two perpendicular passes to ensure even distribution. Control plots received the same amount of water without added N. The 20 L of water used for urea application served only as a carrier solution and was not included in the precipitation treatment.

Precipitation was manipulated by approximately ±50% of ambient throughfall using transparent rain-exclusion shelters [14] and supplemental irrigation. In the W−50% and NW−50% treatments, six transparent plastic shelters (measuring 1 m × 3 m × 1.6 m with over 90% light transmittance) were placed in each subplot at an 8–10° angle to block about half of the rainfall. The intercepted water was collected through grooves and channeled via PVC pipes to nearby W+50% or NW+50% subplots. Shelters and pipes were cleaned monthly.

To monitor soil and litter dynamics, soil samples and litter bags were collected throughout the 2024 growing season. Every two months (May, July, and September), six soil cores (2.5 cm in diameter) were taken from the 0–10 cm layer near the PVC collars in each subplot, removed of visible roots and plant debris, and combined into a single composite sample per subplot. One subsample was air-dried for physicochemical analyses, and another was stored at −20 °C for microbial analyses. Simultaneously, to assess litter decomposition, 30 g of mixed litter from dominant plant species was placed in nylon mesh bags (1 mm mesh size) and distributed throughout all treatment plots. Six litter bags were placed in each subplot to account for potential loss or damage [15]. Litter bags were deployed at the beginning of the 2024 growing season (April 2024) and retrieved at 60, 120, and 180 days (approximately May, July, and September 2024), coinciding with the soil respiration measurement period.

### 2.3. Measurement of Soil Respiration and Its Components

The trenching method was used to split total soil respiration (Rs) into heterotrophic respiration (Rh) and autotrophic respiration (Ra). In each subplot, one area remained undisturbed, while the other was trenched to a depth of 1 m and a width of 60 cm. Visible roots were removed, and nylon mesh was placed around the trenched soil to stop fine-root re-entry. Six PVC collars (15 cm diameter, 10 cm tall) were installed in each subplot at 3 m intervals, with three collars in untrenched soil and three in trenched soil (108 collars total). The collars were inserted 5 cm into the soil.

Respiration measurements began after the treatment-establishment period, allowing the effects of trench-induced root decomposition to stabilize. Measurements were taken weekly during the 2024 growing season (April–September 2024), which is the site’s main biologically active period, when soil temperature usually exceeds 10 °C, most annual rainfall occurs, and nitrogen is applied. CO_2_ efflux from trenched soil was recorded as Rh, and Ra was calculated as the difference between untrenched and trenched soil efflux [14,16]. Prior to each field measurement campaign, the LI-8100A infrared CO_2_/H_2_O gas analyzer was zeroed and spanned against certified standard CO_2_ gas (400 ppm; LI-COR Biosciences, Lincoln, NE, USA) following the manufacturer’s calibration protocol, to ensure measurement accuracy across the growing season. Soil respiration was measured on sunny days between 09:00 and 15:00 using an LI-8100 infrared gas analyzer (LI-COR, Lincoln, NE, USA), following standard chamber measurement protocols to minimize pressure artefacts and exclude post-rain CO_2_ flush events [17]. This mid-day measurement window provides a reliable proxy for daily mean flux by avoiding extreme diurnal temperature fluctuations.

To meet the independence assumption of ANOVA, respiration data were grouped at the subplot level. First, the three collar measurements within each subplot were averaged for each sampling date to prevent pseudo-replication within subplots. Then, these date-level means were averaged across the 2024 growing season to produce a single seasonal mean per subplot for Rs, Rh, and Ra. As a result, the final dataset included 18 independent subplot-level seasonal means (6 treatments × 3 replicates). Soil temperature at 10 cm depth was measured next to each collar using an LI-8100-201 thermocouple probe, and volumetric soil moisture at 5 cm depth was assessed with a portable TDR-200 probe (Spectrum Technologies Inc., Plainfield, IL, USA).

### 2.4. Measurement of Soil Properties

Soil physicochemical and biological properties were assessed from composite soil samples collected in May, July, and September. Soil pH was measured in a 1:2.5 soil-to-water suspension using a pH meter. Soil organic matter was determined using the Walkley–Black wet oxidation method [18]. Available phosphorus was extracted with 0.5 M NaHCO_3_ (Olsen method) and measured colorimetrically [19]. Ammonium (NH_4_^+^) and nitrate (NO_3_^−^) were extracted with 2 M KCl and analyzed with an autoanalyzer. Microbial biomass carbon (MBC) and microbial biomass nitrogen (MBN) were measured using the chloroform fumigation–extraction method [20,21], with a correction factor of kEC = 0.45 for MBC and kEN = 0.54 for MBN applied to fumigated–unfumigated differences [21]. Extracts were analyzed for carbon via a TOC analyzer and for nitrogen using an autoanalyzer.

To determine fine-root biomass (FRB; roots < 2 mm diameter), three additional soil cores (9.3 cm diameter, 0–10 cm depth) were collected from each subplot in May, July, and September 2024. Roots were separated by washing through a 0.5 mm mesh sieve, cleaned of soil and debris, and oven-dried at 65 °C for 48 h until a constant mass was reached. For graphical presentation and between-treatment comparison, FRB values are expressed as the percentage change relative to the control (CK), calculated from seasonal mean dry masses across the three collection dates.

### 2.5. Statistical Analysis

Statistical analyses were performed using R 4.3.2. Normality and homogeneity of variance were assessed with the Shapiro–Wilk and Levene’s tests, respectively. Data were transformed as needed to meet model assumptions, and significance was set at *p* < 0.05. The subplot was considered the independent experimental unit. To prevent spatial and temporal pseudoreplication, the three collar measurements within each subplot were averaged for each sampling date, and these date-level averages were then combined across the 2024 growing season to produce one seasonal mean per subplot for Rs, Rh, and Ra. Consequently, the final analysis included 18 independent seasonal means (6 treatments × 3 replicates).

The effects of nitrogen addition (N), precipitation treatment (W), and their interaction (N × W) on Rs, Rh, and Ra were analyzed using two-way ANOVA. Other biotic and abiotic variables were examined with one-way ANOVA followed by Tukey’s HSD test when appropriate. Relationships between respiration components and environmental variables were analyzed using linear and exponential regression. Temperature sensitivity (Q_10_) was estimated from the exponential model $Rs={a\cdot e}^{bT}$, where $Q10=e^{10b}$. Principal component analysis (PCA) was used to reduce the dimensionality of soil variables. Soil available nutrients (SAN) were calculated as the sum of available phosphorus (AP), ammonium (NH_4_^+^), nitrate (NO_3_^−^), and soil organic carbon (SOC) concentrations and expressed in mg kg^−1^.

Structural equation modelling (SEM) was conducted using IBM AMOS (Analysis of Moment Structures) version 24.0 (IBM Corp., Armonk, NY, USA) using the 18 subplot-level seasonal means to assess the direct and indirect effects of nitrogen addition and precipitation manipulation on Rh and Ra. The model included the following mediator variables: soil moisture (volumetric water content at 5 cm depth), soil temperature (at 10 cm depth), soil pH, microbial biomass carbon (MBC), and fine-root biomass (FRB < 2 mm diameter). Candidate pathways were determined based on PCA-informed variable selection and prior ecological theory. Model fit was evaluated using χ^2^/df < 3, RMSEA < 0.040, and CFI > 0.95, and standardized total effects of all five mediator variables on both Rh and Ra were extracted following [22].

## 3. Results

### 3.1. Abiotic and Biotic Factors

**Figure 1:**
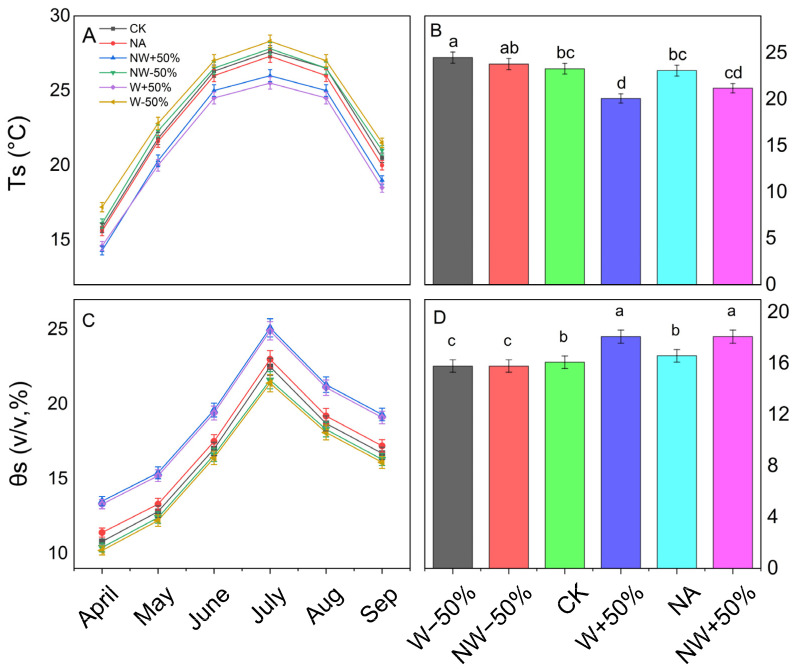
Seasonal variations and average soil temperature (Ts) at 10 cm depth and volumetric water content (θs) at 5 cm depth were examined under different treatments. Panels (**A**,**B**) display temperature data, while Panels (**C**,**D**) show water content data. Treatments include CK (control), NA (nitrogen addition), W+50% (50% increased precipitation), W−50% (50% decreased precipitation), NW+50% (NA with +50% precipitation), and NW−50% (NA with −50% precipitation). Data are shown as mean ± standard error. Significant differences between treatments are marked by lowercase letters.

During the study period, soil temperature at 10 cm depth showed significant seasonal fluctuations, ranging from 15.8 °C in April to a peak of 28.3 °C in July (Figure 1A,B), with notable differences among treatments (*p* < 0.001, Table S1). Compared to the control (CK), the W−50% treatment significantly increased soil temperature by about 5.2%, while the W+50% and NW+50% treatments decreased soil temperature by 13.7% and 9.0%, respectively (*p* < 0.05). The NW−50% treatment also caused a significant increase of 2.1%, whereas nitrogen addition alone (NA) resulted in a slight, non-significant decrease of 0.9% (*p* > 0.05).

The soil volumetric water content (VWC) varied with the seasons, ranging from 7.51% to 27.80% (Figure 1C). The W+50% and NW+50% treatments maintained significantly higher VWC levels (18.1%) than the control (16.1%), while the W−50% and NW−50% treatments reduced VWC to 15.8% (*p* < 0.01). The NA treatment did not produce a statistically significant change in VWC compared to the control (*p* > 0.05).

**Figure 2:**
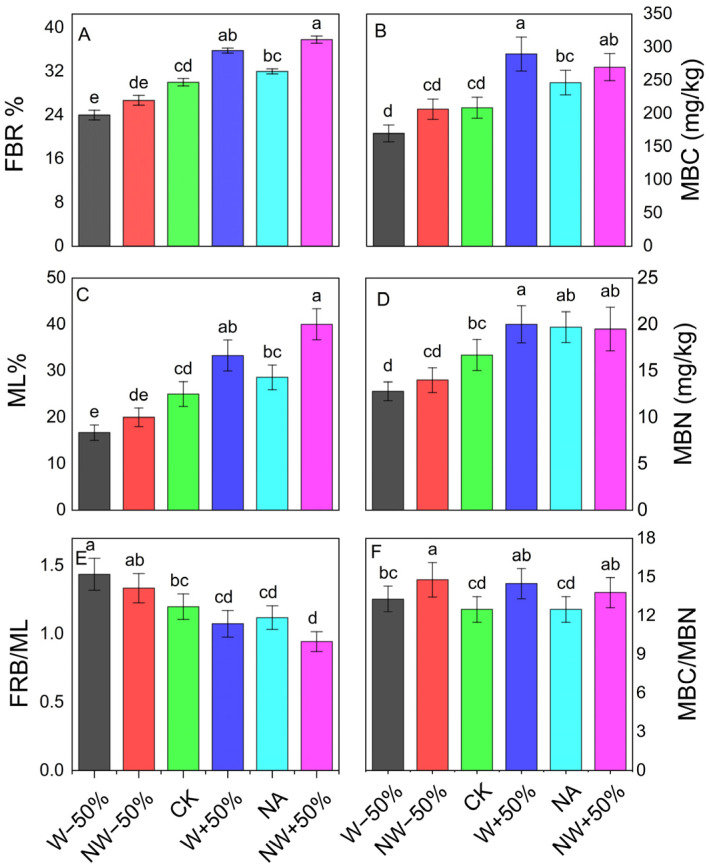
Effects of nitrogen addition and altered precipitation on (**A**) fine-root biomass (FRB, % relative to CK), (**B**) microbial biomass carbon (MBC), (**C**) litter mass loss (ML), (**D**) microbial biomass nitrogen (MBN), (**E**) FRB/ML ratio, and (**F**) MBC/MBN ratio during the 2024 growing season. Significant differences between treatments are marked by lowercase letters.

The experimental treatments significantly affected the soil biotic properties (Figure 2). Nitrogen addition (NA) increased soil ammonium (NH_4_^+^) and nitrate (NO_3_^−^) levels by 28.6% and 35.1%, respectively, compared to the control (*p* < 0.05; Figure S2A,C). Microbial biomass carbon (MBC) was greatly influenced by the treatments (*p* < 0.001). Compared to the control, MBC increased by 38.9% under W+50%, 29.3% under NW+50%, and 18.2% under NA, but decreased by 18.5% under W−50% (Figure 2B). Microbial biomass nitrogen (MBN) showed a similar pattern (*p* < 0.001), increasing by 19.8% under W+50%, 18.0% under NA, and 16.8% under NW+50%, while decreasing by 23.4% under W−50% compared to the control (Figure 2D). The MBC/MBN ratio was also notably affected by the treatments (*p* < 0.001). The biggest increase was observed under NW−50% (18.4%), followed by W+50% (16.0%), with no significant differences among the other treatments (Figure 2F).

Fine root biomass (FRB) was highly responsive to changes in precipitation (*p* < 0.05). The W+50% and NW+50% treatments increased FRB by 19% and 26%, respectively, while W−50% decreased FRB by 20%. In contrast, NA had no significant effect (Figure 2A). Litter mass loss (ML) also showed a strong response to precipitation management. W−50% reduced ML by 33%, while W+50% and NW+50% increased ML by 33% and 60%, respectively (*p* < 0.001). No significant differences were observed in NA and NW−50% compared to the control (*p* > 0.05; Figure 2C).

Soil pH varied significantly among the treatments (*p* < 0.001; Figure S3). Nitrogen addition consistently caused acidification, leading to lower pH levels in the NA, NW−50%, and NW+50% treatments (all *p* < 0.001 compared to control). In contrast, W−50% increased the pH, while W+50% caused a slight but non-significant decrease.

### 3.2. Soil Respiration and Its Components

**Figure 3:**
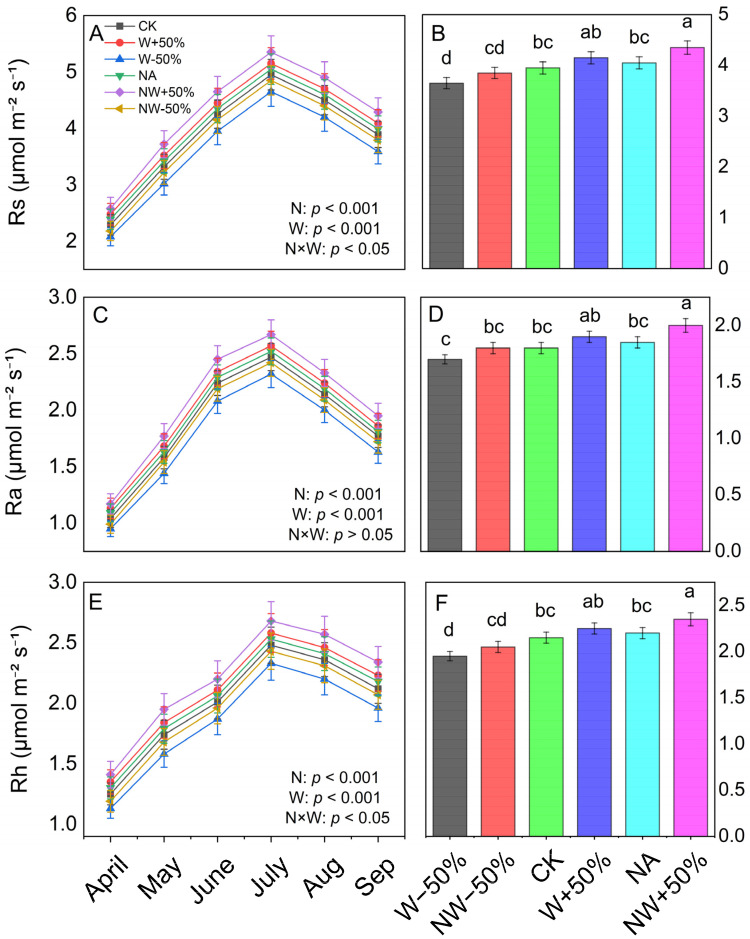
Seasonal dynamics (**A**,**C**,**E**) and seasonal mean values (**B**,**D**,**F**) of total soil respiration (Rs), heterotrophic respiration (Rh), and autotrophic respiration (Ra) under six treatments during the 2024 growing season. Treatments: CK (control), NA (nitrogen addition), W+50% (increased precipitation), W−50% (reduced precipitation), NW+50% (N + increased precipitation), NW−50% (N + reduced precipitation). Data are mean ± standard error (*n* = 3). Different lowercase letters indicate significant differences among treatments (*p* < 0.05). Two-way ANOVA results for nitrogen (N), precipitation (W), and their interaction (N × W) are shown in each panel.

Total soil respiration (Rs) exhibited clear seasonal variation, with values ranging from 2.08 to 5.35 μmol CO_2_ m^−2^ s^−1^, and was significantly affected by both nitrogen input and changes in precipitation (*p* < 0.001) (Figure 3A; Table S1). The NW+50% treatment yielded the highest Rs, showing a 47.1% increase compared to the control (CK). In contrast, W−50% resulted in a notable 7.6% decrease (*p* < 0.05). Throughout the year, Rs peaked in July (4.95–5.35 μmol CO_2_ m^−2^ s^−1^) and reached its lowest in April (2.08–2.58 μmol CO_2_ m^−2^ s^−1^).

Both heterotrophic respiration (Rh) and autotrophic respiration (Ra) showed patterns similar to those of Rs. The NW+50% treatment led to the greatest increases in both fluxes compared to CK (Rh 45.5%, Ra 50.0%; *p* < 0.05) (Figure 3D,F). Two-way ANOVA indicated significant interaction effects of nitrogen and precipitation on Rs and Rh (*p* < 0.05), but Ra did not show a significant interaction (*p* > 0.05; Table 1). Post hoc comparisons further showed that, under drought conditions, N addition still stimulated Rs, Rh, and Ra compared to drought alone (NW−50% vs. W−50%), increasing by 31.5%, 36.4%, and 28.7%, respectively (*p* < 0.05; Figure 3), although this stimulation was significantly weaker than that observed under elevated precipitation.

Under ambient precipitation, nitrogen addition (NA vs. CK) significantly increased Rs, Rh, and Ra by 11.8%, 12.1%, and 11.1%, respectively (*p* < 0.05), consistent with the significant main effect of N in the two-way ANOVA (Table 1; *p* < 0.001 for all three components). The strongest effect was observed under increased precipitation, where nitrogen addition combined with increased precipitation (NW+50% vs. CK) increased Rs, Rh, and Ra by 47.1%, 45.5%, and 50.0%, respectively (*p* < 0.001; Figure S4).

**Figure 4:**
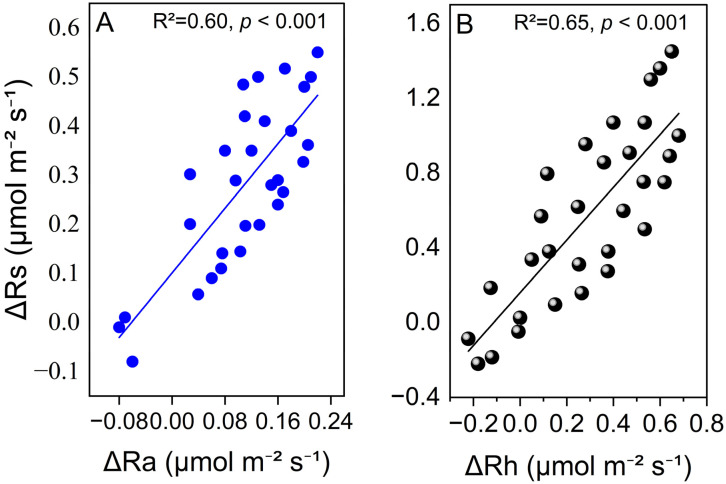
Relationships between treatment-induced changes in total soil respiration (ΔRs) and changes in (**A**) heterotrophic respiration (ΔRh) and (**B**) autotrophic respiration (ΔRa) during the 2024 growing season. Δ values = treatment mean − control (CK) mean. R^2^ and *p*-values are shown for each regression.

Across all treatments, Rh consistently contributed 53–56% of the total Rs. Nitrogen addition generally increases the relative contribution of heterotrophic processes. For example, under W+50%, the Rh/Rs ratio increased from 0.52 to 0.54 (Figure S5). The largest absolute increase in Rs compared to CK was observed in NW+50% (ΔRs = 1.70 ± 0.20 μmol CO_2_ m^−2^ s^−1^), while the W−50% treatment caused a significant decrease (ΔRs = −0.28 ± 0.04 μmol CO_2_ m^−2^ s^−1^). Regression analyses showed that variation in Rs was more strongly associated with ΔRh (R^2^ = 0.65, *p* < 0.001) than with ΔRa (R^2^ = 0.60, *p* < 0.001; Figure 4A,B), indicating a slightly greater influence of heterotrophic processes on treatment-related changes in total soil respiration. However, because the difference in explanatory power was small, this pattern should be interpreted with caution.

### 3.3. Relationships Between Environmental Variables and Soil Respiration

**Figure 5:**
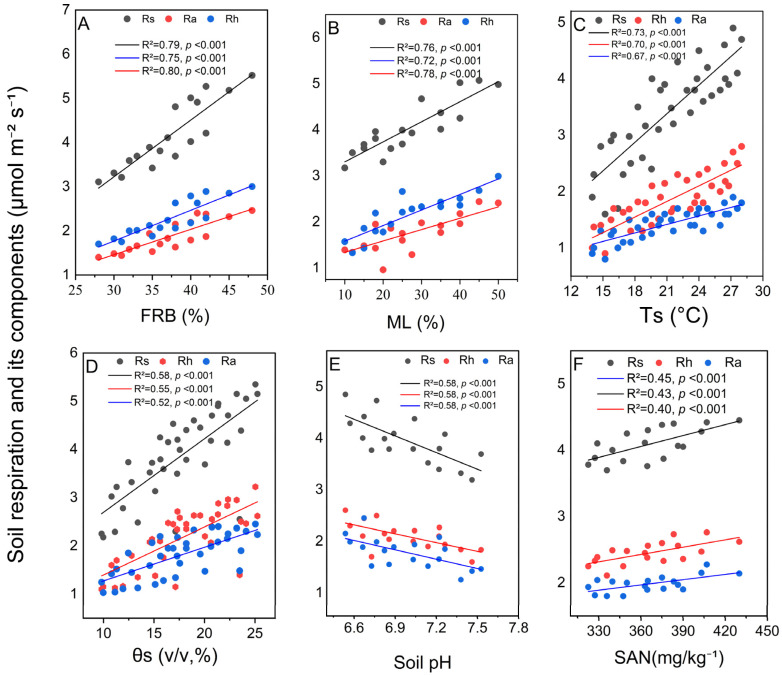
Relationships between soil respiration components (Rs, Rh, Ra) and biotic and abiotic factors during the 2024 growing season. Panels (**A**,**B**) show relationships with fine-root biomass (FRB) and litter mass loss (ML), respectively. Panels (**C**,**D**) display relationships with soil temperature (Ts, at 10 cm depth) and volumetric soil water content (θs, at 5 cm depth), respectively. Panels (**E**,**F**) illustrate relationships with soil pH and soil available nutrients (SAN = z-score composite of AP, NH_4_^+^, NO_3_^−^, and SOC), respectively. R^2^ and *p*-values are provided for each regression line. Significant treatment differences are marked with lowercase letters.

Soil respiration (Rs) and its components were significantly affected by both abiotic and biotic factors. Rs showed a positive relationship with soil temperature (R^2^ = 0.82), as did Rh (R^2^ = 0.85) and Ra (R^2^ = 0.78) (Figure 5C). The temperature sensitivity (Q_10_) of Rs, Rh, and Ra, calculated from exponential relationships with soil temperature, is shown in Table S2. Soil volumetric water content also had a positive correlation with Rs, Rh, and Ra, with R^2^ values of 0.58, 0.55, and 0.52, respectively (Figure 5D). In contrast, respiration rates were negatively correlated with soil pH, with Rs, Rh, and Ra all displaying significant negative relationships; the R^2^ value for Rs was 0.58 (Figure 5E).

Biotic attributes had equally significant influences. Fine root biomass (FRB) and litter mass loss (ML) were particularly strong predictors of Rs, with explanatory powers of R^2^ = 0.79 and R^2^ = 0.76, respectively (*p* < 0.001) (Figure 5A,B). Soil available nutrients (SAN = mean of AP, NH_4_^+^, NO_3_^−^, and SOC concentrations; mg kg^−1^) showed positive trends with all respiration components in regression analysis (Figure 5F), although this relationship was not statistically significant (*p* > 0.05), which aligns with the lack of a direct soil nutrient–respiration pathway in the SEM. Available phosphorus alone showed a similar non-significant trend (Figure S2B; Table S3).

**Figure 6:**
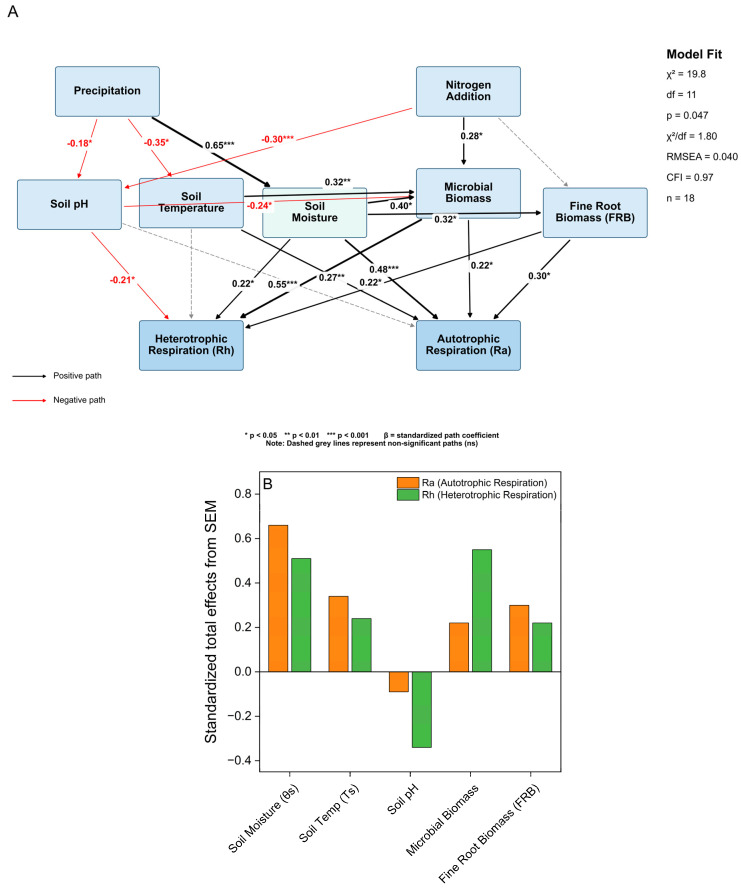
Structural equation model (SEM) showing the direct and indirect effects of nitrogen addition and precipitation manipulation on soil respiration components during the 2024 growing season (*n* = 18 subplot-level seasonal means). (**A**) Rectangles represent observed variables. Solid black arrows indicate positive significant paths, red arrows indicate negative significant paths, and grey dashed arrows represent non-significant paths (ns). * *p* < 0.05; ** *p* < 0.01; *** *p* < 0.001. Model fit: χ^2^ = 19.8, df = 11, *p* = 0.047, χ^2^/df = 1.80, RMSEA = 0.040, CFI = 0.97, *n* = 18. (**B**) Standardized total effects of all five mediator variables on Ra and Rh extracted from the SEM in panel (**A**).

Structural equation modelling (SEM) revealed both direct and indirect pathways through which nitrogen addition and precipitation influence soil respiration components (Figure 6A). Precipitation significantly increased soil moisture (β = 0.65, *p* < 0.001) and independently decreased soil pH (β = −0.18, *p* < 0.05), while nitrogen addition further lowered soil pH (β = −0.30, *p* < 0.001). Nitrogen addition also directly enhanced microbial biomass (β = 0.28, *p* < 0.05), aligning with the 18.2% increase in MBC observed under the NA treatment (Figure 2B).

Soil moisture positively affected microbial biomass (β = 0.40, *p* < 0.05), fine root biomass (FRB) (β = 0.32, *p* < 0.05), and directly influenced both autotrophic respiration (β = 0.48, *p* < 0.001) and heterotrophic respiration (β = 0.22, *p* < 0.05). Soil temperature had a significant positive effect on Ra (β = 0.27, *p* < 0.01) and also significantly influenced microbial biomass (β = 0.32, *p* < 0.05), thereby indirectly affecting Rh. Soil pH negatively impacted Rh (β = −0.21, *p* < 0.05) and microbial biomass (β = −0.24, *p* < 0.05), while its direct effect on Ra was non-significant. Microbial biomass was the main direct driver of Rh (β = 0.55, *p* < 0.001) and also directly influenced Ra (β = 0.22, *p* < 0.05). Fine root biomass (FRB) was a significant driver of Ra (β = 0.30, *p* < 0.05) and also directly contributed to Rh (β = 0.22, *p* < 0.05). Nitrogen addition had no significant direct effect on fine root biomass (FRB) (β = 0.05, ns). No direct paths from soil nutrients to Ra or Rh were included, as SAN showed no significant correlation with respiration components (r = 0.075, *p* > 0.05).

## 4. Discussion

Our findings show that water availability mainly determines how nitrogen (N) deposition affects soil carbon fluxes in this temperate forest in North China [4,5]. We observed a strong interactive effect in which nitrogen addition and changes in precipitation levels jointly influenced the direction and strength of ecosystem responses. Specifically, under increased precipitation, nitrogen addition increased total soil respiration (Rs) and its heterotrophic (Rh) and autotrophic (Ra) components. Under reduced precipitation, nitrogen addition still stimulated Rs, Rh, and Ra compared to drought alone (NW−50% vs. W−50%), although the stimulation was significantly weaker than under increased precipitation (Figure 3 and Figure S4). Notably, Rh was more sensitive to the nitrogen × precipitation interaction than Ra (Table 1). Structural equation modelling (SEM) further revealed that these patterns were driven by different biotic and abiotic pathways (Figure 6). Overall, these results emphasise water availability as a key “switch” controlling ecosystem responses to N inputs and highlight the importance of including such interactions in models predicting forest carbon cycling under global change [5].

### 4.1. Effects of N Addition on Rs and Its Components

Consistent with findings from other N-rich temperate ecosystems [23], nitrogen addition under ambient precipitation significantly increased Rs by 11.8% (*p* < 0.05; NA vs. CK; Table 1), along with corresponding increases in its components. This relatively modest response likely reflects a balance between the positive effects of N addition on microbial activity and the negative impact of N-induced soil acidification, which can suppress enzyme activity and change microbial community composition.

The significant increase in autotrophic respiration (Ra) (+11.1%) aligns with the carbon allocation stability framework in plants [24,25]. According to the optimal resource allocation framework, plants increase root production only when doing so improves acquisition of the most limiting resource [26]. Our results showed that N addition had no significant effect on fine root biomass (FRB) (Figure 2A), indicating that nitrogen was not the primary limiting factor for plant growth. Instead, productivity in this ecosystem is likely co-limited by other nutrients, such as phosphorus, or more critically by water availability, as our findings suggest [13,27]. SEM analysis supported this conclusion, as the direct pathway from N addition to fine root biomass (FRB) was not significant (β = 0.05, ns; Figure 6). Overall, carbon allocation to root maintenance and function, and therefore Ra, remained unchanged because there was no significant change in the total root biomass pool, which was identified as an important axis of variability in PCA (PC3).

The notable increase in heterotrophic respiration (Rh) (+12.1%) also indicates a balance between opposing microbial processes under N addition treatment [28]. A well-documented inhibitory pathway of chronic N enrichment, supported by our data, is N-induced soil acidification. We observed a significant decline in soil pH within the N addition plots (Figure S3), and SEM confirmed a negative causal relationship between N addition and soil pH (β = −0.30) (Figure 6). Soil pH plays a crucial role in regulating microbial community composition, enzyme activity, and metabolic efficiency [8]. Acidification can directly impair microbial function, and previous studies have shown that it is a primary mechanism behind reduced microbial activity during N deposition [29,30]. Therefore, it is plausible that any stimulatory effects of added N on microbial metabolism are offset by the inhibitory effects of soil acidification on microbial metabolism [30]. This interplay of opposing drivers may account for the variable microbial responses often reported under N enrichment, as increased acidity can inhibit certain microbial processes while stimulating others, leading to inconsistent results. Overall, our findings suggest that in N-rich temperate forests, the soil carbon cycle remains resilient against moderate N inputs, demonstrating considerable resistance to moderate N enrichment [31,32]. Mechanistically, this buffering likely results from microbial functional redundancy, where N-sensitive taxa are replaced by N-tolerant ones, maintaining overall metabolic rates. Additionally, N addition simultaneously stimulates anabolic (biomass production) and catabolic (decomposition) microbial processes, so that net CO_2_ efflux at the ecosystem level shows only modest change.

### 4.2. Effects of Altered Precipitation on Rs and Its Components

Our results showed that altered precipitation alone did not significantly affect total soil respiration (Rs) or its components (Figure 3). This finding contrasts with many previous studies reporting strong drought-induced reductions in soil carbon fluxes in temperate ecosystems [33,34]. We interpret this apparent stability as reflecting complex ecosystem dynamics, where resistance to hydrological stress, along with compensatory adjustments in biotic pools and physiological processes, conceals the expected drought effects. The temperate forest studied here, characterised by deep soils and large organic matter reserves, is likely more resistant to moderate hydrological stress than shallower-rooted systems, such as grasslands [35]. This resilience probably enables roots and microbial communities to access deeper soil water, maintaining baseline respiration rates even under decreased precipitation [29,36].

Although overall Rs remained relatively stable, drought clearly altered its underlying biotic controls. The W−50% treatment reduced both fine root biomass (FRB) and microbial biomass carbon (MBC) (Figure 2A,B), indicating suppression of the two primary biological contributors to soil respiration [1,37]. Microbial biomass nitrogen (MBN) also decreased significantly under drought (Figure 2D), further suggesting that water limitation caused physiological stress on microbial communities and restricted their growth and survival [38].

The elevated MBC/MBN ratio under W−50% supports increased fungal dominance or greater allocation to carbon-rich osmolytes as an adaptation to drought stress [31]. Drought also reduced available phosphorus (Figure S2B), indicating secondary nutrient limitation caused by decreased mineralization and diffusion processes [39]. Interestingly, heterotrophic respiration (Rh) remained stable despite the decline in microbial biomass, suggesting that the stress-tolerant microbial community had higher mass-specific respiration rates [29,36]. Therefore, the apparent stability of Rs under altered precipitation conditions should not be interpreted as ecosystem resistance, but rather as an emergent property of a stressed system where surviving organisms maintain function at higher energy costs [29,40]. Mechanistically, soil water potential controls water film thickness around soil particles, which in turn influences substrate diffusion to microbial cells and extracellular enzyme mobility. Under moderate drought, fungal hyphae sustain connectivity across drying soil microsites, supporting enzymatic decomposition and heterotrophic CO_2_ efflux. This fungal hydraulic continuity likely explains why Rh remained stable despite reduced MBC under W−50% in this forest.

### 4.3. Interaction Between N Addition and Altered Precipitation

The interaction between nitrogen (N) addition and precipitation was the main factor affecting soil respiration (Rs) in our study. This provides strong evidence for the idea of resource co-limitation, which suggests that ecosystem responses to one resource are limited by the availability of other resources [41]. Our data show that the effect of N on Rs was consistently stimulatory across moisture conditions, but its magnitude was heavily influenced by water availability, emphasizing the importance of examining multiple global change drivers simultaneously in future studies. This finding aligns with results from other water-limited ecosystems [12,42] and highlights the limitations of single-factor experimental designs in predicting ecosystem responses [43].

Under increased precipitation, N addition synergistically increased Rs, Rh, and Ra, with Rs rising by 47.1% (Figure S4). Our SEM analysis confirmed that precipitation strongly increased soil moisture, which in turn boosted microbial biomass, improved fine root biomass (FRB), and directly raised both Ra and Rh. Soil temperature contributed independently to Ra, while N-induced and precipitation-induced soil acidification suppressed Rh. Ultimately, microbial biomass remained the primary causal driver of Rh, confirming that the heterotrophic pathway is mainly controlled by biological rather than direct abiotic factors in this ecosystem (Figure 6).

Enhanced plant productivity under these conditions likely increased microbial activity through rhizosphere priming, in which higher root exudation of labile carbon stimulated microbial metabolism and accelerated the breakdown of soil organic matter, thereby supporting Rh [2,44]. This response was accompanied by a noticeable decline in surface soil organic carbon (SOC, 0–10 cm) under the NW+50% treatment (Figure S2D).

The observed decline in surface SOC aligns with increased litter decomposition and higher carbon emissions. However, this finding is limited to the 0–10 cm soil layer and does not reflect the carbon dynamics of the entire soil profile or the broader ecosystem. Therefore, these results should not be regarded as evidence of a widespread shift from a carbon sink to a carbon source.

Under drought conditions, N addition continued to boost respiration compared to drought alone, although at a significantly lower level than in wet conditions. This moisture-dependent change in the N effect supports the idea of resource co-limitation: when water is the primary limiting factor, extra N produces only minor improvements in biological activity because root and microbial functions are already hindered by low soil moisture. Soil moisture affects substrate diffusion, enzyme reaction rates, and the delivery of soluble nitrogen to microbial cells [45]. Therefore, even though N inputs increased soil nutrient availability during drought, microbes and roots could not fully utilize this additional N due to insufficient water. The decline in MBN observed under NW−50% (Figure 2D) reflects water-limited microbial activity, and any positive influence of N on Rh during drought was necessarily constrained by the reduced microbial biomass pool [28].

A key insight is the different sensitivity of the Rs components to this interaction. While both microbial and root biomass decline under drought, Rh remains, suggesting that stress-adapted microbes compensate through increased metabolic activity, whereas root respiration shows no such resilience [13,46]. Consistent with this, ANOVA identified a significant N × precipitation interaction for Rh (*p* < 0.05), but not for Ra (*p* > 0.05) (Table 1). This divergence arises from differences in how carbon sources are used. Autotrophic respiration depends on recent photosynthate, and drought quickly reduces belowground carbon allocation due to stomatal closure [25,46]. In contrast, heterotrophic respiration is driven by the decomposition of existing organic matter, a process that is heavily limited by soil moisture’s effect on substrate diffusion and enzyme activity [47,48]. Therefore, the higher sensitivity of Rh demonstrates its stronger reliance on soil physical conditions. These differences highlight the importance of explicitly modelling microbial and root respiration as separate processes in carbon–climate feedback systems [12,49].

These findings have wider implications for how carbon–climate feedbacks respond to simultaneous N deposition and changes in precipitation. Increased N and moisture lead to the simultaneous stimulation of Ra and Rh, indicating faster soil carbon turnover—more inputs from root exudates and litter, combined with increased microbial decomposition—from which net carbon storage could decrease even as gross primary productivity increases. This two-way acceleration is not represented by models that treat Rs as a single, unified flux. Therefore, it is essential to include the mechanistic separation of Ra and Rh, along with their distinct biological controls (root biomass for Ra; microbial biomass and soil pH for Rh), into Earth system models to improve the accuracy of projections for the carbon balance in temperate forests under future global change.

## 5. Conclusions

Nitrogen’s effect on soil respiration in this temperate forest was strongly affected by water availability. Nitrogen addition significantly raised Rs under normal rainfall (+11.8%, *p* < 0.05) and further increased Rs, Rh, and Ra under higher rainfall, probably because of increased fine root biomass and enhanced litter breakdown. During drought conditions, N addition still boosted Rs, Rh, and Ra compared to drought alone, but the effect was much weaker than with increased precipitation. These results show that N addition consistently enhances soil carbon fluxes across different moisture levels, but water availability greatly influences the extent of N stimulation. They highlight the importance of considering interactive N–water effects when forecasting forest carbon fluxes and suggest that rainfall patterns greatly affect ecosystem responses to elevated nitrogen deposition. These findings may also improve the parameterisation of N–water interactions in Earth system models. However, interpretation should be cautious due to the single-site, single-season approach, limitations of FRB and SOC measurements to the top 0–10 cm, and the absence of non-growing-season respiration data.

## Figures and Tables

**Table 1:** Results (*p*-values) of two-way ANOVA showing the effects of nitrogen addition (N), altered precipitation (W), and their interactions on soil total respiration (Rs), heterotrophic respiration (Rh), and autotrophic respiration (Ra).

Source of Variation	Rs	Rh	Ra
Nitrogen (N)	<0.001	<0.001	<0.001
Precipitation (P)	<0.001	<0.001	<0.001
N × P Interaction	<0.05	<0.05	>0.05

## Data Availability

The original contributions presented in this study are included in the article/Supplementary Materials. Further inquiries can be directed to the corresponding author.

## Supplementary Materials

The following supporting information can be downloaded at: https://www.mdpi.com/article/10.3390/plants15091340/s1.
