# Supplementary material for: The Interactive Effect of Rainfall and Nitrogen Deposition on Soil Respiration and Its Components in a Temperate Forest Ecosystem
Source: Plants (Basel). 2026 Apr 28;15(9):1340. doi: 10.3390/plants15091340 (PMC13164911; doi:10.3390/plants15091340)
Supplement: Supplementary file 1 [file plants-15-01340-s001.zip › plants-4197826-supplementary.pdf]

Supplementary data

Fig.S1-S5

Fig.S1: A map of the study site and a diagram of the experimental design.

Fig.S2: The effects of nitrogen and precipitation treatments on soil nutrients.

Fig.S3: The impact of experimental treatments on soil pH.

Fig.S4: Relative changes in soil respiration components induced by nitrogen addition across different precipitation levels.

Fig.S5: The contribution of heterotrophic respiration to total soil respiration under various nitrogen and precipitation treatments.

Supplementary Tables

Tables S1-S3

Table S1: A summary of the effects of nitrogen and precipitation treatments on various soil abiotic and biotic properties.

Table S2: Regression equations showing the sensitivity (Q10) of soil respiration components to soil temperature.

Table S3: Results from the principal components analysis (PCA) for soil nutrients, microbial biomass, and plant activity indicators.

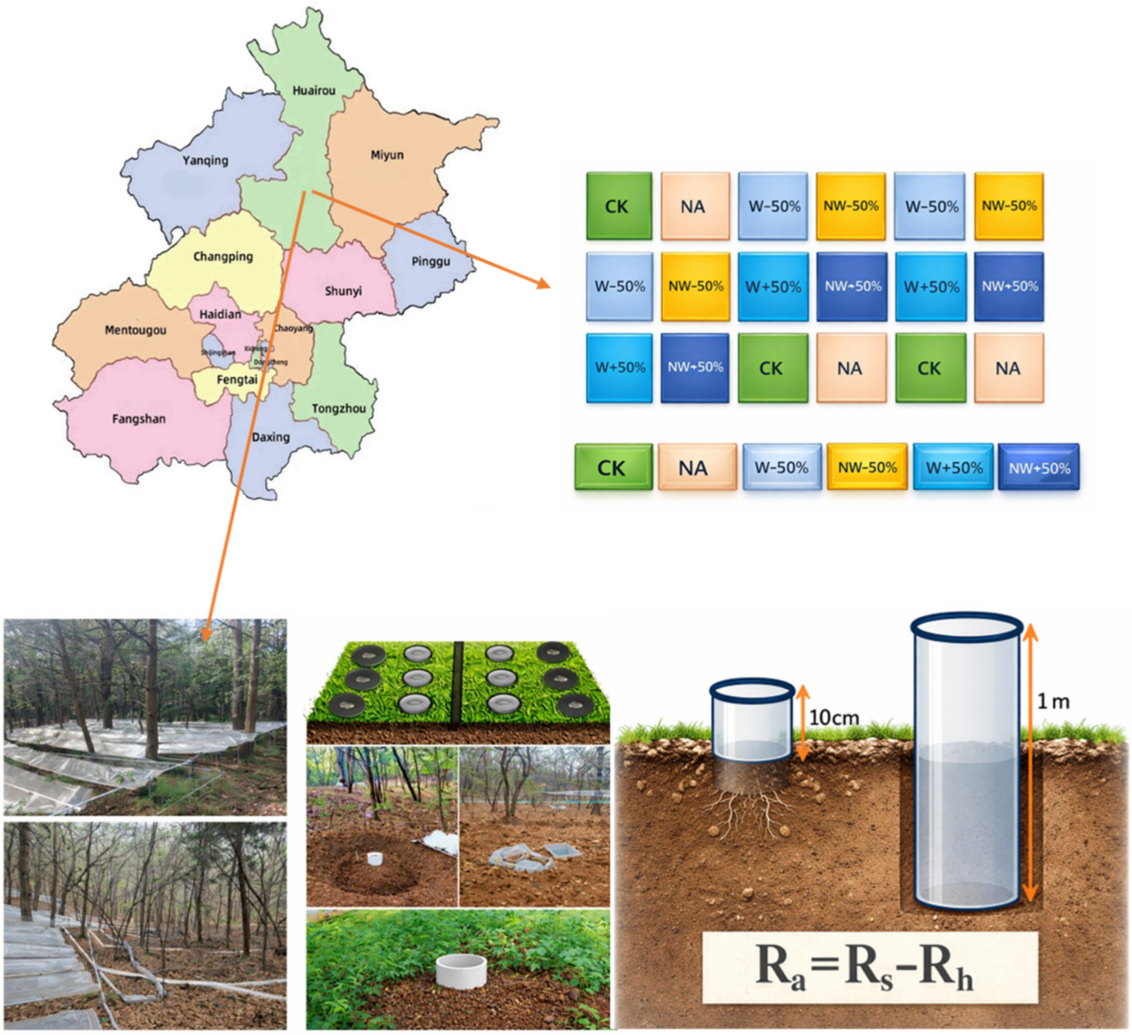

Fig. S1 Study site and experimental design in a temperate forest ecosystem in Huairou, Beijing. Experimental treatments include CK (control), N (nitrogen addition), W+50% (50% increased precipitation), W-50% (50% decreased precipitation), NW+50% (nitrogen addition with 50% increased precipitation), and NW-50% (nitrogen addition with 50% decreased precipitation).

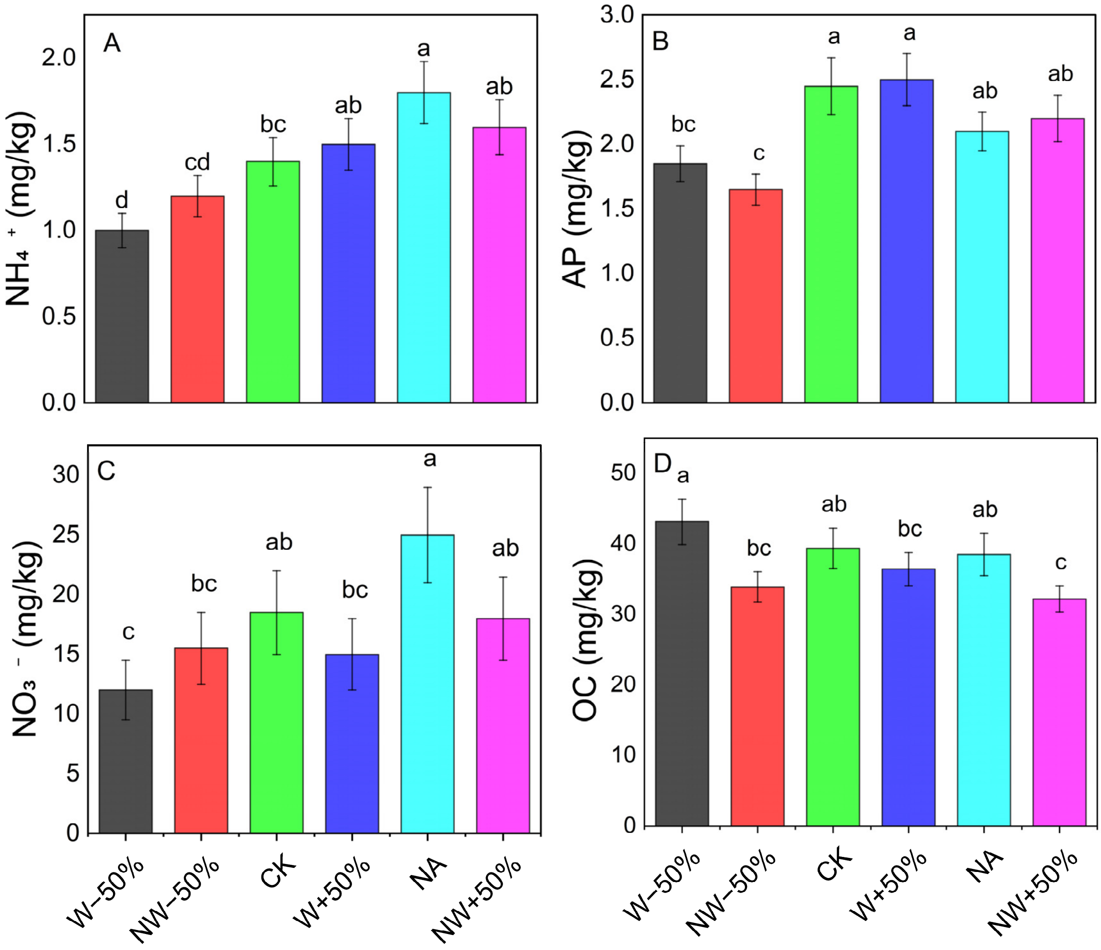

Fig. S2 Effects of nitrogen addition and altered precipitation on soil ammonium (NH_4^+-N) (A), available phosphorus (AP) (B), nitrate (NO_3^--N) (C), and soil organic carbon (SOC) (D). Lowercase letters above the bars indicate significant differences among treatments ($P < 0.05$). Error bars represent the standard error of the mean (n = 3).

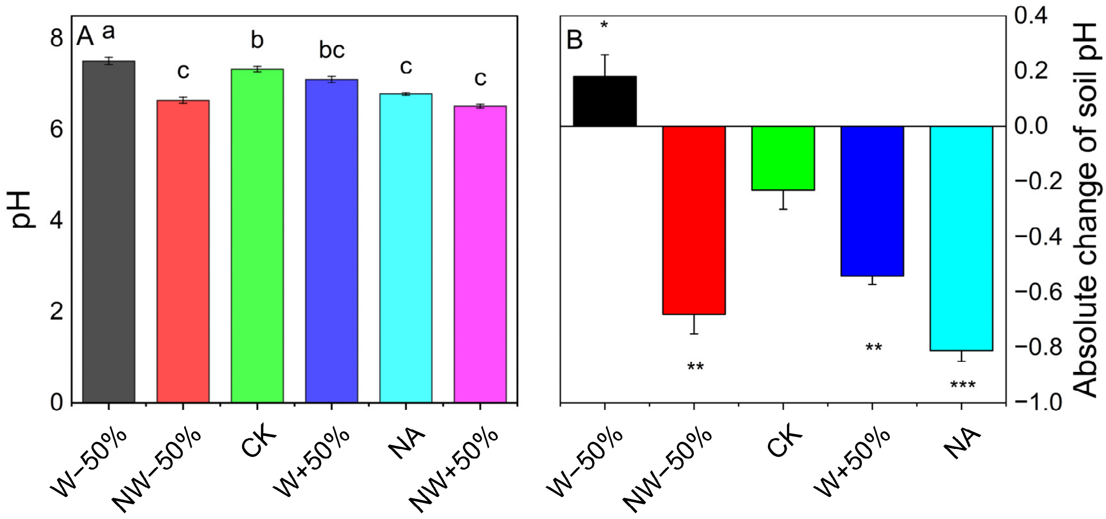

Fig. S3 The effect of nitrogen addition and altered precipitation on the average soil pH and the absolute changes observed. Data marked with *, **, and *** indicate significant differences at P < 0.05, P < 0.01, and P < 0.001, respectively. Treatments include N (nitrogen addition), W+50% (50% increased precipitation), W−50% (50% decreased precipitation), NW+50% (nitrogen + 50% increased precipitation), and NW−50% (nitrogen + 50% decreased precipitation). Error bars show the standard error of the mean (n = 3).

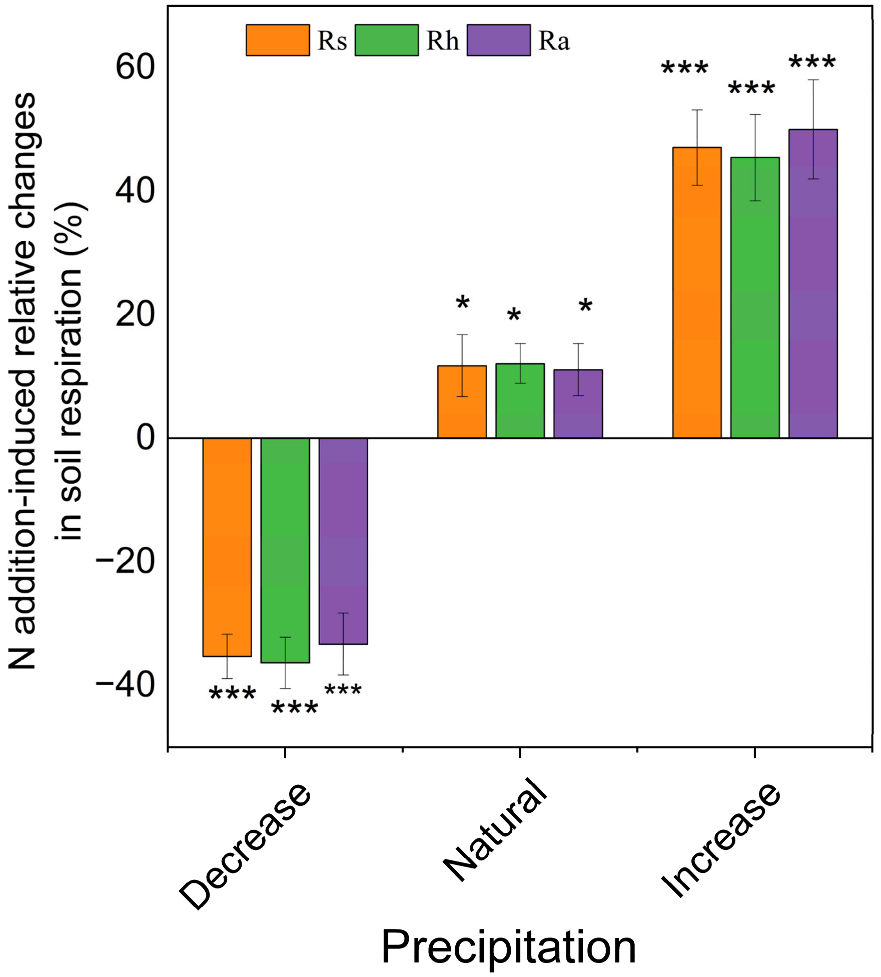

Fig. S4 N addition-induced relative changes (RN1-RN0)/RN0×100, %) in soil respiration (Rs, Rh, and Ra) under different precipitation levels (decreased, natural, increased) in 2024. RN1 represents soil respiration with N addition, and RN0 represents soil respiration without N addition. Bars marked with *, **, and *** indicate significant differences at $P < 0.05$, $P < 0.01$, and $P < 0.001$, respectively. Vertical bars represent standard errors of the mean.

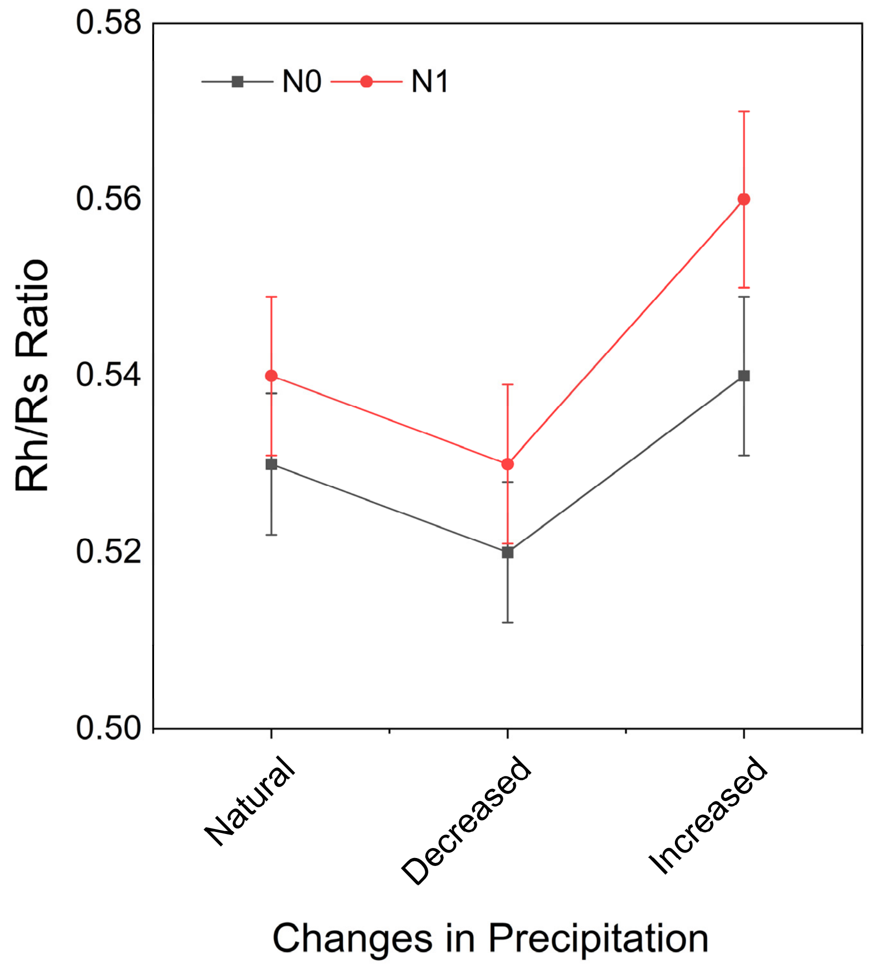

Fig. S5 Contribution of heterotrophic respiration to total soil respiration (Rh/Rs ratio) in plots with and without nitrogen (N) addition under three precipitation treatments during the 2024 growing season. Bars represent mean ± standard error (SE, n = 6).

Table S1 Effects of nitrogen (N) and precipitation (P) treatments on soil abiotic and biotic properties. Values are presented as mean ± standard error. Different lowercase letters indicate significant differences between treatments ($p < 0.05$). Treatments include CK (control), NA (N addition), W+50% (50% precipitation increase), W-50% (50% precipitation decrease), NW+50% (N addition with 50% precipitation increase), and NW-50% (N addition with 50% precipitation decrease).

**Parameter**	**CK**	**NA**	**W+50%**	**W-50%**	**NW+50%**	**NW-50%**
Abiotic Factors						
Soil Temperature (°C)	23.3 ± 0.58 ^{bc}	23.1 ± 0.58 ^{bc}	20.1 ± 0.50 ^{d}	24.5 ± 0.60 ^{a}	21.2 ± 0.50 ^{cd}	23.8 ± 0.60 ^{ab}
Soil Moisture, θs (%)	16.1 ± 0.49 ^{b}	16.6 ± 0.49 ^{b}	18.1 ± 0.51 ^{a}	15.8 ± 0.48 ^{c}	18.1 ± 0.51 ^{a}	15.8 ± 0.48 ^{c}
Soil pH	7.33 ± 0.06 ^{b}	6.79 ± 0.03 ^{c}	7.10 ± 0.07 ^{bc}	7.51 ± 0.08 ^{a}	6.52 ± 0.04 ^{c}	6.65 ± 0.07 ^{c}
Soil Properties						
Organic Carbon, OC (g kg^{-1})	39.40 ± 2.86 ^{ab}	38.54 ± 2.98 ^{ab}	36.45 ± 2.34 ^{bc}	43.15 ± 3.21 ^{a}	32.22 ± 1.87 ^{c}	33.92 ± 2.15 ^{bc}
Available Phosphorus, AP (mg kg^{-1})	2.45 ± 0.22 ^{a}	2.10 ± 0.15 ^{ab}	2.50 ± 2.20 ^{a}	1.85 ± 0.14 ^{bc}	2.20 ± 0.18 ^{ab}	1.65 ± 0.12 ^{c}
Ammonium Nitrogen, NH_4^+-N (mg kg^{-1})	1.40 ± 0.14 ^{bc}	1.80 ± 0.18 ^{a}	1.50 ± 0.15 ^{ab}	1.00 ± 0.10 ^{d}	1.60 ± 0.16 ^{ab}	1.20 ± 0.12 ^{cd}
Nitrate Nitrogen, NO_3^--N (mg kg^{-1})	18.5 ± 3.5 ^{ab}	25.0 ± 4.0 ^{a}	15.0 ± 3.0 ^{bc}	12.0 ± 2.5 ^{c}	18.0 ± 3.5 ^{ab}	15.5 ± 3.0 ^{bc}
Microbial Biomass Carbon, MBC (mg kg^{-1})	208.7 ± 15.67 ^{cd}	246.6 ± 18.67 ^{bc}	289.7 ± 25.67 ^{a}	170.0 ± 12.67 ^{d}	269.9 ± 20.33 ^{ab}	206.6 ± 15.33 ^{cd}
Microbial Biomass Nitrogen, MBN (mg kg^{-1})	16.7 ± 1.67 ^{bc}	19.7 ± 1.67 ^{ab}	20.0 ± 2.00 ^{a}	12.8 ± 1.00 ^{d}	19.5 ± 2.33 ^{ab}	14.0 ± 1.33 ^{cd}
Plant & Decomposition						
Fine Root Biomass (%)	30.0 ± 0.67 ^{cd}	32.0 ± 0.47 ^{bc}	35.8 ± 0.47 ^{ab}	24.0 ± 0.88 ^{e}	37.8 ± 0.67 ^{a}	26.7 ± 0.88 ^{de}

Mass Loss (%)	25.0 ± 2.67 [cd]	28.6 ± 2.67 [bc]	33.3 ± 3.33 [ab]	16.7 ± 1.67 [e]	40.0 ± 3.33 [a]	20.0 ± 2.00 [de]

Table S2 Regression equations and sensitivity (Q10) of soil respiration (Rs, Rh, Ra) to soil temperature (Ts) during the growing season.

Respiration Component	Regression Equation	R^2	*p*-value	Q_{10}
Total (R_s)	$R_s = 0.76e^{0.068Ts}$	0.82	< 0.001	1.97
Heterotrophic (R_h)	$R_h = 0.39e^{0.073Ts}$	0.85	< 0.001	2.07
Autotrophic (R_a)	$R_a = 0.37e^{0.063Ts}$	0.78	< 0.001	1.88

Table S3 Component matrix of principal components analysis (PCA) for soil nutrients (OC, available P, NH_4^+-N, NO_3^--N), soil microbial biomass (MBC, MBN), and plant activity indicators (FRB%, ML%).

Soil Nutrients PC1	Loading	Microbial Biomass PC2	Loading	Plant Activity PC3	Loading
SOC	0.80	MBC	0.85	FRB%	0.85
Available P	0.75	MBN	0.80	ML%	0.80
NH_4^+-N	0.70				
NO_3^--N	0.65				
Proportion Explained	43.60%	Proportion Explained	22.70%	Proportion Explained	8.20%
